# Functional Changes of the Perigenual Part of the Anterior Cingulate Cortex after External Trigeminal Neurostimulation in Migraine Patients

**DOI:** 10.3389/fneur.2017.00282

**Published:** 2017-06-15

**Authors:** Antonio Russo, Alessandro Tessitore, Fabrizio Esposito, Federica Di Nardo, Marcello Silvestro, Francesca Trojsi, Rosa De Micco, Laura Marcuccio, Jean Schoenen, Gioacchino Tedeschi

**Affiliations:** ^1^Headache Center, Department of Medical, Surgical, Neurological, Metabolic and Aging Sciences, University of Campania “Luigi Vanvitelli”, Naples, Italy; ^2^MRI Research Center SUN-FISM, University of Campania “Luigi Vanvitelli”, Naples, Italy; ^3^Department of Medicine, Surgery and Dentistry “Scuola Medica Salernitana”, University of Salerno, Baronissi, Italy; ^4^Liège University, Headache Research Unit, University Department of Neurology, Citadelle Hospital, Liège, Belgium

**Keywords:** migraine, BOLD, functional magnetic resonance, imaging, fMRI, Cefaly^®^

## Abstract

**Objective:**

To explore the functional reorganization of the pain processing network during trigeminal heat stimulation (THS) after 60 days of external trigeminal neurostimulation (eTNS) in migraine without aura (MwoA) patients between attacks.

**Methods:**

Using whole-brain BOLD-fMRI, functional response to THS at two different intensities (41 and 51°C) was investigated interictally in 16 adults MwoA patients before and after eTNS with the Cefaly^®^ device. We calculated the percentage of patients having at least a 50% reduction of monthly migraine attacks and migraine days between baseline and the last month of eTNS. Secondary analyses evaluated associations between BOLD signal changes and clinical features of migraine.

**Results:**

Before eTNS treatment, there was no difference in BOLD response between MwoA patients and healthy controls (HC) during low-innocuous THS at 41°C, whereas the perigenual part of the right anterior cingulate cortex (ACC) revealed a greater BOLD response to noxious THS at 51°C in MwoA patients when compared to HC. The same area demonstrated a significant reduced BOLD response induced by the noxious THS in MwoA patients after eTNS (*p* = 0.008). Correlation analyses showed a significant positive correlation between ACC BOLD response to noxious THS before eTNS treatment and the decrease of ACC BOLD response to noxious THS after eTNS. Moreover, a significant negative correlation in the migraine group after eTNS treatment between ACC functional activity changes and both the perceived pain ratings during noxious THS and pre-treatment migraine attack frequency has been found.

**Conclusion:**

Our findings suggest that eTNS treatment with the Cefaly^®^ device induces a functional antinociceptive modulation in the ACC that is involved in the mechanisms underlying its preventive anti-migraine efficacy. Nevertheless, further observations to confirm whether the observed fMRI effects of eTNS are both related to clinical improvement and specific to antinociceptive modulation in migraine patients are mandatory.

## Introduction

Migraine is the most prevalent neurological disorder worldwide and ranked sixth among the leading causes of years lived with disability (1). Unfortunately, migraine is not curable and most preventive pharmacotherapies are characterized by a relatively low efficacy and disabling adverse effects. There is thus a need for more effective and better tolerated treatments. Recently, based on new insights in migraine and pain pathophysiology, non-invasive neurostimulation therapies have raised much interest (2) Among them, interventions targeting pericranial nerves have become part of the preventive armamentarium (3).

In particular, external trigeminal neurostimulation (eTNS) with the Cefaly^®^ device has demonstrated a very favorable efficacy/safety profile in migraine patients (4), when compared with that of preventive anti-migraine drugs (5), although comparisons with other neuromodulation approaches are lacking, to date. Besides in a randomized controlled clinical trial (4), the beneficial effects of eTNS for migraine prevention were confirmed in a large survey (6) and in a recent open-label trial (7). More recently, a fluorodeoxyglucose-positron emission tomography (FDG-PET) study showed that eTNS with the Cefaly^®^ device was able to increase activity in crucial areas of the limbic system and salience matrix such as orbitofrontal (OF) and anterior cingulate cortex (ACC) in migraine patients responding to the treatment (8). This suggests that eTNS may be able to modulate neuronal circuits involved in the descending pain control of trigeminovascular nociceptors in the spinal trigeminal nucleus (3). Direct proof, however, of an eTNS-induced change in trigeminal nociceptive processing is lacking. We decided therefore to explore with whole-brain BOLD fMRI the functional reorganization of the pain processing network during trigeminal heat stimulation (THS) after 60 days of eTNS with the Cefaly^®^ device in migraine without aura (MwoA) patients between attacks.

We hypothesized that eTNS would induce a functional reorganization of the trigeminal pain-processing network and increase activity of central antinociceptive mechanisms in MwoA patients.

## Materials and Methods

### Subjects

We enrolled 20 patients suffering from MwoA according to the criteria of the International Classification of Headache Disorders (ICHD-3 beta version, code 1.1) (9) with a low frequency of attacks (≤5 attacks/month). Ten of these patients had previously participated in our study exploring the clinical efficacy of eTNS (6). All patients were right-handed and had a normal neurological examination. The patients had never taken migraine-preventive drugs in the course of their life. Exclusion criteria were the presence of any other ICHD-III diagnosis (e.g., tension type headache, chronic migraine, etc.), somatic or psychiatric conditions, or intake of daily medications. Only patients compliant with the eTNS therapy were included in the final analysis. Compliance was defined as use of the device for ≥800 min during the 60-day treatment period (i.e., ≥2/3 of the total time expected) and according to neurostimulation pattern of preventive parameters. Based on this criterion, two patients were excluded from the final statistical analysis. Moreover, two additional patients were excluded due to movement artifacts during the fMRI scan. To avoid any possible attack- or treatment-related interference, patients were pain free during the fMRI recording and had not taken any acute migraine drug for at least 3 days before the scanning. They were interviewed by telephone 3 days after the MRI scan to ascertain that they were also migraine free during this period.

Sixteen right-handed subjects with a comparable age- and sex distribution were recruited as healthy controls (HC) *via* advertisements placed in the hospital (e.g., posters and flyers), *via* word-of-mouth referrals, and from a database of research volunteers maintained by the MRI Research Center of the Second University of Naples (Table 1). All subjects underwent preliminary MRI examination before entering the present study.

**Table 1:** Demographic characteristics of migraine patients and HC and clinical characteristics of migraine patients before and after eTNS.

Parameter	Group	Mean ± SE	*p* Value
Gender	MwoA	15 F/1 M	
	HC	15 F/1 M	
Age (years)	MwoA	31.31 ± 2.33	0.35
	HC	29.13 ± 1.60	
Disease duration (years)		8.3 ± 1.7	
Monthly migraine attack frequency	Baseline	4.25 ± 0.23	<0.001
	After eTNS	1.87 ± 0.27	
Monthly migraine day frequency	Baseline	6.31 ± 0.41	<0.001
	After eTNS	3.25 ± 0.45	
Monthly NSAID intake (including acetaminophen)	Baseline	3.06 ± 0.61	0.01
	After eTNS	1.25 ± 0.36	
Monthly triptan intake	Baseline	2.25 ± 0.63	0.05
	After eTNS	0.87 ± 0.29	
Total intake of abortive medications	Baseline	5.31 ± 0.37	<0.001
	After eTNS	2.12 ± 0.31	
HIT-6	Baseline	61.81 ± 1.35	<0.001
	After eTNS	52.69 ± 1.35	
Attack intensity (VAS)	Baseline	7.78 ± 0.10	0.01
	After eTNS	6.6 ± 0.24	

*F, female; M, male; NSAID, non-steroidal anti-inflammatory drugs; HIT-6, Headache Impact Test; VAS, Visual Analog Scale; MwoA, migraine without aura; HC, healthy controls; eTNS, external trigeminal neurostimulation*.

### Standard Protocol Approvals, Registrations, and Patients’ Consent

The experiments conformed to the principles of the Declaration of Helsinki and were approved by the ethics committee of the Second University of Naples. All participants provided informed, written consent after the experimental procedure had been explained.

### eTNS Treatment and Clinical Outcome Measures

The study was conducted between January 2013 and February 2015.

After a 28-day baseline without preventive treatment, patients applied daily during 20 min the Cefaly^®^ device for 60 days. They were instructed how to apply the device and to use stimulation protocol 2 (pulse frequency 60 Hz, max intensity 16 mA) and returned to the clinic for a final evaluation at the end of the eTNS treatment period. An in-built electronic system allowed to monitor the time of use of the device and the correct parameters of neurostimulation for each patient (see above).

Frequency of migraine attacks and migraine days per month, mean pain intensity during attacks using the VAS score (0: no pain; 10: severe pain totally prohibiting daily activities) and impact on daily life (HIT-6 score) (10) as well as monthly intake of rescue medication were measured with paper headache diaries during a 28-day pre-treatment baseline and during the 60 days of eTNS with the Cefaly^®^ device (Table 1). Clinical features such as disease duration and disability (MIDAS score) were obtained from the patients when they entered the study. A migraine day was defined as a day with headache fulfilling ICHD-III beta version criteria for MwoA (9), except for duration, if the attack was treated. Migraine days not separated by at least one headache-free day were considered to belong to the same migraine attack. We measured the percentage change in headache parameters between baseline and the last month of eTNS and calculated the percentage of patients having at least a 50% reduction of monthly migraine attacks and migraine days.

JMP software (Version 11, SAS Institute Inc., NC, USA) was used to perform the statistical analysis on the clinical data. Continuous data were expressed as means ± SEs and compared using the paired *t*-test or Wilcoxon matched pairs signed-rank test where appropriate. Statistical significance was defined as *p* < 0.05.

### Trigeminal Heat Stimulation

Heat stimuli were applied using the contact heat-evoked potential stimulator (CHEPS) (Medoc Ltd, Ramat Yishai, Israel). The CHEPS device has an MRI-compatible thermode with an area of 572.5 mm^2^ and a heating thermo-foil (Minco Products, Inc., Minneapolis, MN, USA), covered with a 25 mm layer of thermo conductive plastic (Kapton^®^, thermal conductivity at 23°C of 0.1–0.35 W/m/K). It is characterized by a rapid rising time to high temperatures (up to 70°C/s) suited to study thermonociceptive pathways. Patients were tested on the cheek on the side more frequently affected during migraine attacks, whereas HC were matched with regard to the side of the face tested in migraine patients. We compared a low innocuous stimulus at 41°C (11, 12) and a noxious stimulus at 51°C (11, 12). To minimize the effects of habituation and expectation, all experimental stimuli were delivered at random.

### Functional Magnetic Resonance Imaging Parameters and Pre-Processing

The fMRI imaging parameters and pre-processing used in the present study were described in detail previously (11, 12). Briefly, MRI was performed on a 3-T scanner (Signa HDxt, GE Healthcare, USA) equipped with an eight-channel parallel head coil. Each fMRI scan consisted of 300 volumes of a repeated gradient-echo echo planar imaging sequence. Three-dimensional T1-weighted images (FSPGR BRAVO sequence) and T2-fluid-attenuated inversion recovery sequence was also acquired in all subjects.

Functional image time-courses were processed using the software package BrainVoyager QX (Brain Innovation, The Netherlands). All the scans were re-aligned to the first included volume scan using a Levenberg–Marquardt algorithm in order to correct for movement artifacts. Then, the motion parameters were carefully inspected to control that no excessive residual motion (>1 functional voxel) was present. Using the results of the image registration with three-dimensional anatomical scans, the functional image time series were warped into Talairach space and resampled into 3-mm isotropic voxel time series. Finally, to perform a group-level analysis, the resampled volume time series were spatially filtered (smoothing) using a 6-mm full-width-at-half-maximum Gaussian kernel.

### Experimental Protocol

Migraine patients, before eTNS treatment, and HC underwent two consecutive fMRI sessions for each of the two THS stimuli (41 and 51°C) according to a previously reported event-related experimental design (11, 12). In each fMRI session, 600 ms THS was applied to the maxillary skin at two different temperatures (41 and 51°C) with a jittered inter-stimulus interval of 14 ± 1 s (total session duration 7 min 45 s). Prior to the fMRI recordings, outside the scanner, migraine patients and HC were fully informed about the characteristics of the applied thermal heat stimuli. After each fMRI session, there was a delay of about 30 s during which subjects, inside the scanner, had to verbally rate the perceived pain induced by the heat stimulus on a numerical rating scale (NRS) ranging from 0 (“no pain”) to 10 (“worst pain imaginable”).

After the 60-day eTNS treatment migraine patients underwent an additional fMRI session similar to the pre-treatment session described above.

### Statistical Analysis

The description of statistical analysis used in the present study was described in detail previously (11, 12). The variance of all image time series was estimated voxel-wise according to a random-effects convolution-based general linear model analysis (13). For each subject, the two fMRI time series corresponding to the two separate sessions were temporally normalized to *z* scores and concatenated before entering the general linear model (GLM) fitting (see below). Two “event-type” GLM predictors of interest encoding the responses to the two stimulus types (41 and 51°C) were defined using the double-gamma function as hemodynamic input function for the linear convolution. For each subject and each voxel included in the slab of imaging, the “beta” weights of all regressors were estimated according to a GLM fit–refit procedure.

To draw population-level inferences from statistical maps, the two beta estimates for the predictors of interest at each voxel entered a second-level analysis of variance with subjects treated as random observations (random effects analysis of variance ANOVA). Two ANOVA models were calculated: in the first model, at each voxel, a two-way ANOVA table was calculated, with one within-subject factor for the “temperature” effect and one between-subject factor for the MwoA effect; in the second model, at each voxel, a two-way ANOVA table was calculated with one within-subject factor for the “temperature” effect and one within-subject factor for the eTNS treatment in MwoA patients.

From the computed ANOVA tables, contrast t-maps for the main effects of THS as well as for the two-group and one-group (MwoA before treatment > MwoA after treatment) differential effects were computed and overlaid on the Talairach-normalized high-resolution “Colin-27” template.

To localize the regions with statistically significant effects, a threshold was applied to the t-maps, which protected against false-positive voxels at 5% (corrected for multiple comparisons). In order to correct functional clusters for multiple comparisons, a cluster-level threshold was applied to the maps that protected against false-positive clusters at 5% (cluster-level corrected for multiple comparisons). More specifically, starting from an (uncorrected) voxel-level threshold of *p* = 0.005, a whole-brain correction approach based on Monte Carlo simulations was used to define the minimum cluster size (14).

To test for possible eTNS effects in the group of MwoA patients as well as for correlations between regional BOLD responses to THS and clinical scores (i.e., disease duration, migraine frequency, average of pain intensity of migraine attacks, MIDAS and HIT-6), a region of interest (ROI) was functionally defined from clusters exhibiting statistically significant group effects in the voxel-based analysis and ROI-GLM estimates for the predictors of interest were calculated for each MwoA patient and each scan (before and after eTNS). The obtained ROI-GLM estimates were scaled to BOLD percent signal change and both used in one-sample paired *t*-test to statistically assess the effect of eTNS treatment in MwoA patients and correlated with the individual clinical scores. In addition to functionally defined ROIs, an extra ROI was anatomically defined in the pons. We chose the location based on our previous findings in another population of migraineurs (11) as well as on previous neuroimaging observations in migraine patients (15).

## Results

### Clinical Data

**Figure 1:**
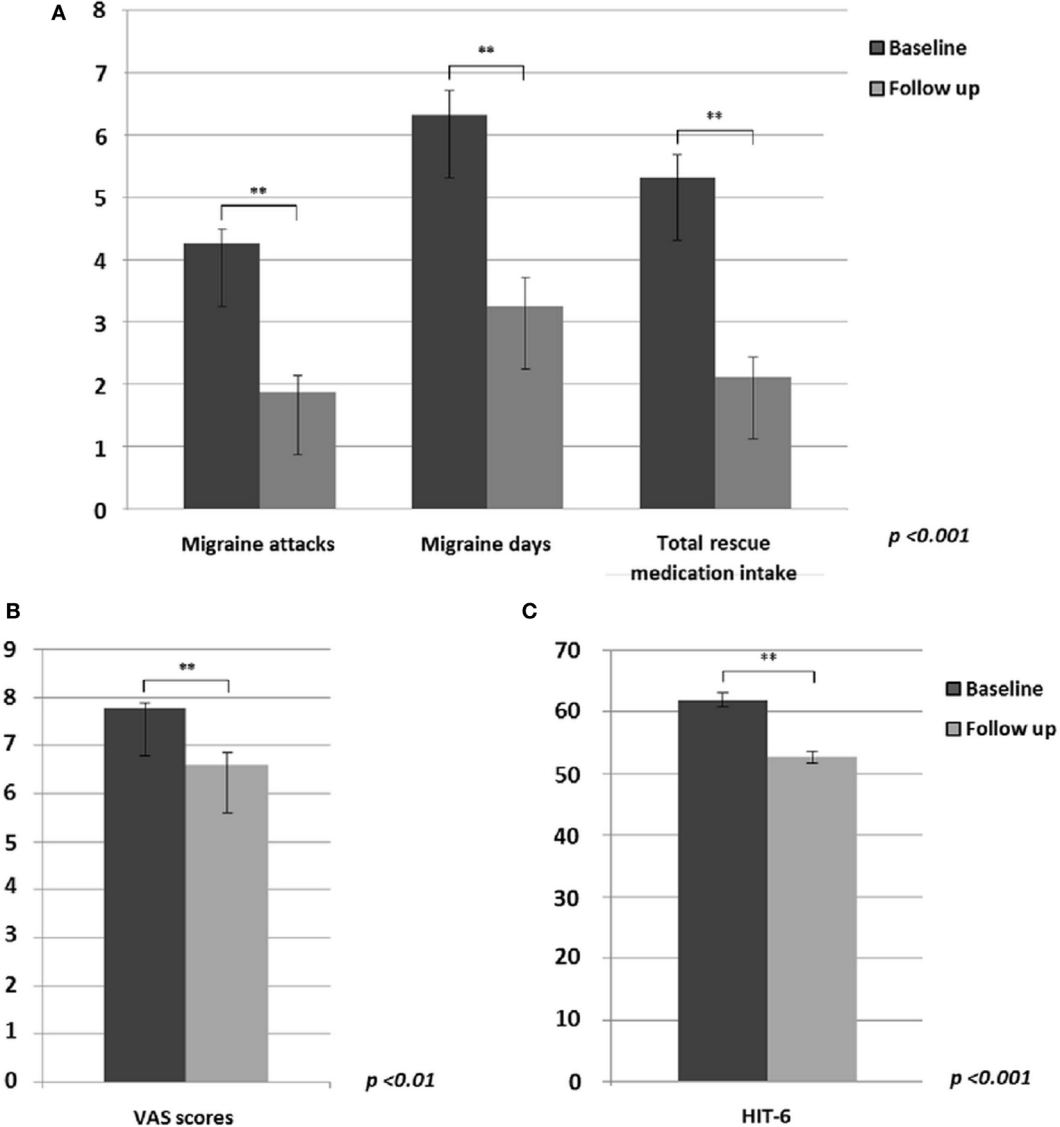
Significant differences between migraine without aura patients before and after external trigeminal neurostimulation treatment in: **(A)** migraine attacks (*p* < 0.001), migraine days (*p* < 0.001) and monthly intake of total rescue medication (*p* < 0.001); **(B)** mean headache severity during migraine attacks (*p* < 0.01); **(C)** HIT-6 questionnaire rating (*p* < 0.001).

Based on patients’ diaries, between baseline and the end of 60-day tSNS treatment (concerning the last month of treatment), we observed a statistically significant decrease in monthly frequency of migraine attacks (*p* < 0.001) and migraine days (*p* < 0.001) and a consequent reduced monthly intake of rescue medication (*p* < 0.001) (Figure 1A). Furthermore, mean pain intensity during attacks (*p* = 0.01) (Figure 1B) and the HIT-6 score (*p* < 0.001) (Figure 1C) were reduced at the end of the treatment. In line with our previous study (6), 81% of patients showed a reduction of at least 50% of migraine attacks and at least 75% of migraine days after eTNS treatment.

### Pain Ratings

Mean intensity of perceived pain (NRS) was not significantly different between MwoA patients before eTNS treatment and HC neither at 41°C (*p* = 0.54) nor at 51°C (*p* = 0.55). Similarly, pain ratings in MwoA patients did not change significantly after eTNS treatment for the 41°C (*p* = 0.33) or the 51°C stimulus (*p* = 0.29).

### Imaging Data

During THS at the two different intensities, functional changes were identified in brain regions known to be involved in pain processing in both groups of subjects as reported in a previous study (11) (*p* = 0.05 cluster level corrected).

During low innocuous THS (41°C), there was no difference in BOLD response between MwoA patients before treatment and HC.

**Figure 2:**
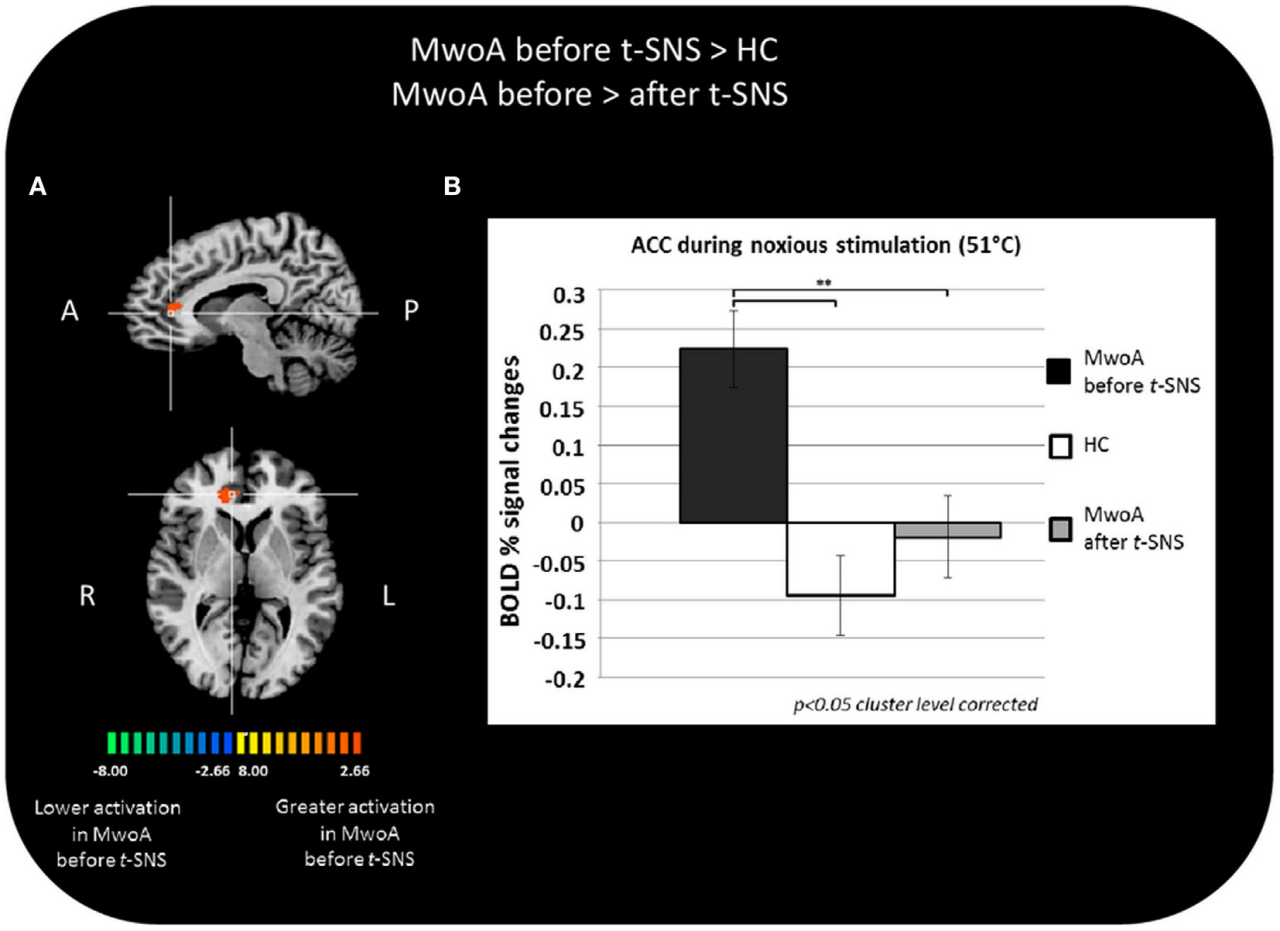
Significant different BOLD response in the group comparisons between patients with migraine without aura (MwoA) before external trigeminal neurostimulation (eTNS) treatment and healthy controls (HC) and between MwoA patients before and after eTNS treatment. **(A)** T-map of statistically significant differences between groups overlaid onto a Talairach transformed Colin-27 T1 high-resolution anatomical template; **(B)** bar graphs of percent BOLD signal changes at Talairach coordinates (*x, y, z*): right anterior cingulate cortex (ACC) = 12, 35, 7 during noxious trigeminal heat stimulation at 51°C in MwoA patients before and after eTNS treatment and HC group.

During noxious THS (51°C), the BOLD response in the perigenual part of the right ACC was significantly greater in MwoA patients before treatment than in HC (Talairach coordinates *x, y, z*: 12, 35, 7; *t*-value (peak) = 4.33) (Figure 2A).

In the same area, we found a significant reduction of the BOLD activation induced by the noxious THS in MwoA patients after eTNS (*p* = 0.008). Mean percentages of BOLD signal changes extracted from the perigenual part of the right ACC in HC and MwoA patients before and after eTNS are shown in Figure 2B.

No significant differences were detected in the whole-brain analysis of eTNS effects in MwoA patients, and no significant regional differences were observed in the pons between-subject groups, conditions or THS temperatures (i.e., MwoA patients compared to HC; MwoA patients before compared to after eTNS treatment).

### Correlation Analyses

**Figure 3:**
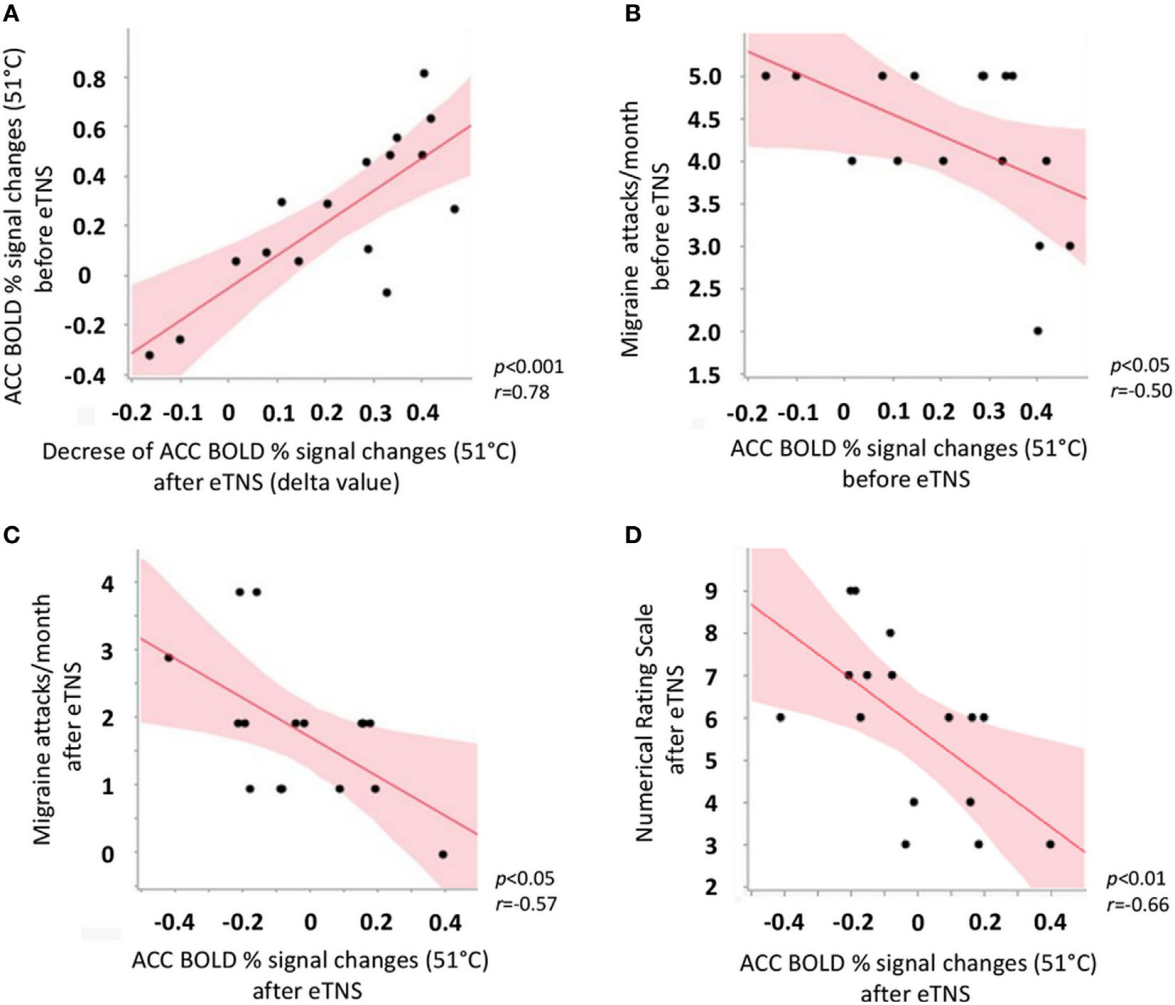
Scatterplot showing significant correlations between **(A)** anterior cingulate cortex (ACC) BOLD response to noxious trigeminal heat stimulation (THS) before external trigeminal neurostimulation (eTNS) treatment and the decrease of ACC BOLD response to noxious THS after eTNS (i.e., the “delta value”); **(B)** ACC BOLD response to noxious THS before eTNS treatment and migraine attack frequency pre-treatment (days/month, y axis); **(C)** ACC BOLD response to noxious THS after eTNS treatment and migraine attack frequency after treatment (days/month, *y* axis); **(D)** ACC BOLD response to noxious THS after eTNS treatment and perceived pain intensity ratings during the THS (numerical rating scale score, *y* axis).

In the migraine group after eTNS treatment, but not in HC, we observed a significant positive correlation between ACC BOLD response to noxious THS before eTNS treatment and the decrease of ACC BOLD response to noxious THS after eTNS (i.e., the “delta value”) (*p* < 0.001; *r* = 0.78). Furthermore, a significant negative correlation was found between BOLD signal changes in ACC before eTNS treatment and migraine attack frequency pre-treatment (*p* < 0.05; *r* = −0.50) as well as between BOLD signal changes in ACC after eTNS treatment and migraine attack frequency after treatment (*p* < 0.05; *r* = −0.57) (i.e., the greater the ACC activation before and after eTNS treatment, the greater the number of migraine attacks per month before and after eTNS treatment). Finally, a significant negative correlation was also found between the perceived pain ratings during THS and fMRI-BOLD signal changes extracted from the ACC cluster, both after eTNS treatment [i.e., the higher the ACC activation after eTNS treatment, the lower the NRS score (*p* = 0.01; *r* = −0.66)] (See Figure 3 for further details).

## Discussion

In the present study, we found that the BOLD response to noxious THS (51°C) was increased in the perigenual ACC in MwoA patients between attacks compared to HC. A 60-day eTNS treatment with the Cefaly^®^ device was able to normalize this abnormal ACC BOLD response and in parallel to significantly improve migraine severity. These results indicate that eTNS induces in MwoA patients a functional reorganization of the trigeminal pain processing network activated by a noxious thermal stimulus and suggests that this might be relevant for its therapeutic effects.

Nowadays, neurostimulation may offer an alternative to pharmacological therapy in migraine, burdened by a number of contraindications and side effects (2, 3). Recently, the PREMICE trial (PREvention of MIgraine using Cefaly), a prospective, multicenter, double-blind, randomized, and sham-controlled trial provided evidence that eTNS is effective in migraine prevention and allows to reduce intake of abortive anti-migraine drugs (4). These data were confirmed by an open-label trial conducted on MwoA patients who experienced a low frequency of migraine attacks and were naïve to preventive anti-migraine drugs (6). Moreover, a retrospective survey in a large cohort of migraine patients using the Cefaly^®^ device (5) evidenced the excellent safety and tolerability of the device.

However, the mechanisms of action underlying eTNS treatment are still largely unknown. The initial rationale for the use of eTNS postulated that convergence of somatic afferents from the trigeminal or the C2 territories with visceral trigeminovascular afferents on spinal trigeminal nucleus nociceptors (16) may block ascending impulses in the pain pathway *via* the phenomenon of “after-suppression” in trigeminal nociceptors (17). This hypothesis was not confirmed in several studies showing that neither eTNS nor per- or transcutaneous ONS decreased the sensitivity of trigeminal nociceptors (18).

On the other hand, evidence has accrued from percutaneous suboccipital neurostimulation (ONS) in chronic cluster headache and chronic migraine suggesting that slow neuromodulatory mechanisms in pain processing brain areas might be related to beneficial therapeutic effects. In particular, a H_2_^15^O-PET study showed significant changes in regional cerebral blood flow in the pons, cuneus, pulvinar and ACC in ONS-treated patients with chronic migraine (19) and a FDG-PET study increased glucose uptake in perigenual ACC in chronic cluster headache patients responding to ONS (20). More recently, a FDG-PET study (8) in episodic migraine patients before and after eTNS with Cefaly^®^ revealed a normalization of the pre-treatment hypometabolism of OF cortex and ACC. Taken together, these data suggest that in patients with primary headaches, ONS might restore the balance within dysfunctioning pain control centres, by modifying top-down activity of pain control, and that the ACC plays a crucial role in this process.

It is well known that the ACC is a key structure involved in analgesia mechanisms as well as in the affective and emotional dimensions of pain, such as self-related negative emotional states (21). The role of ACC in MwoA was also demonstrated in our previous studies showing an increased fMRI BOLD response in this area during noxious THS (11), particularly in patients with ictal allodynia (12). Recently, ^1^H-MRS measurements have shown a “complex” of metabolite alterations in the ACC of migraine patients during the interictal period, supporting its hyperexcitability even between attacks (22).

In line with these findings, our results indicate that the excessive interictal activation of the ACC by noxious THS in MwoA patients normalizes after eTNS treatment, which could be related to its clinical efficacy.

Contrasting with previous studies (11), our brainstem ROI-based analyses did not reveal significant eTNS-induced BOLD response changes in the brainstem of MwoA patients. Diffusion tensor imaging confirmed the presence of anatomical connections between cortical and brainstem pain processing regions (23). These connections enable top-down influences originating in cortical areas and likely modulate pain perception *via* opioid release in the brainstem (24, 25). This is supported by animal experiments showing that transcutaneous electrical nerve stimulation can exert anti-nociception by activating the periaqueductal gray matter (26). We hypothesize that the present negative result may be due to the fact that functional recruitment of brainstem areas in descending modulation is detectable only with higher levels of pain, such as those experienced in the course of more severe painful stimulations or during a migraine attack.

From a clinical point of view, we observed significant improvements in multiple migraine severity parameters after eTNS treatment with the Cefaly^®^ device: a significant decrease in monthly frequency of both migraine attacks and migraine days, in mean pain intensity, and in daily life disability. The percentage of patients with ≥50% reduction of monthly migraine attacks and migraine days was remarkably high compared to available preventive drug therapies. Moreover, eTNS allowed a significant reduction of abortive medication intake, which is of interest for pharmaco-economical reasons and for the prevention of medication overuse headache.

Correlation analysis showed a significant negative correlation between ACC BOLD response to THS and the frequency of attacks both before and after eTNS treatment. These data are apparently in disagreement with the main findings of our study showing, after eTNS treatment, both a reduced ACC BOLD response to THS and a coexistent improvement of several clinical parameters in migraine patients. In this context, our data suggest that the eTNS treatment does not overturn the analgesic role of ACC in migraine patients, characterized by a compensatory increased activity likely in an attempt to reduce the frequency of migraine attacks (11, 12). Contrariwise, the eTNS treatment may produce a re-setting toward lower values of ACC activity (i.e., reduced BOLD response to THS) which is, notwithstanding, involved in the anti-nociception (i.e., the higher the ACC activation, the lower the NRS score after the eTNS treatment) and more evident in patients showing a greater ACC abnormality before the eTNS treatment (i.e., the “delta value”). On the other hand, also a decreased activity of ACC may lead to analgesic mechanism, as previously demonstrated (27). Finally, we cannot exclude that the increased ACC BOLD response to THS before the eTNS and reduced ACC BOLD response to THS after the eTNS may represent an epiphenomenon of migraine attacks frequency (i.e., the higher the frequency of migraine attacks, the higher the ACC activation, both before and after the eTNS treatment).

We are aware that our study has several limitations. First, we did not use an eTNS sham device and, therefore, we cannot rule out the possible role of a placebo effect in imaging and clinical data (28). However, the superiority of effective eTNS respect to sham stimulation for the prevention of migraine headaches has already been demonstrated in a randomized, sham-controlled trial (4). Second, our HC did not undergo eTNS treatment and we can thus not determine if the eTNS-induced changes in ACC activation by THS are specific to migraineurs. By corollary, we cannot exclude that these changes could be due to the clinical improvement of patients after eTNS, rather than to the neurostimulation treatment itself.

Further studies are needed to ascertain that the therapeutic benefit of eTNS in MwoA patients is mediated by the ACC functional normalization.

In conclusion, our results suggest that eTNS treatment with the Cefaly^®^ device may induce a functional antinociceptive modulation in the ACC that may be involved in the mechanisms underlying its preventive anti-migraine efficacy.

We believe that further advances in the comprehension of neurostimulation mechanisms may shed light on migraine pathophysiology, and *vice versa*. Indeed, although compelling evidence supports the hypothesis of a dysfunctional central nervous system in migraine, we confirm that interventions targeting the peripheral nervous system are able to modulate neuronal circuits involved in central sensitization and pain control.

## Ethics Statement

The experiments conformed to the principles of the Declaration of Helsinki and were approved by the ethics committee of the Second University of Naples. All participants provided informed, written consent after the experimental procedure had been explained.

## Author Contributions

AR: study concept and design, data analysis, results interpretation, and manuscript drafting and revision. AT: study concept and design, data analysis, results interpretation, and manuscript revision. FE: fMRI data analysis and results interpretation. FN: fMRI data analysis. MS: data analysis and results interpretation. FT: fMRI data analysis and results interpretation. RM: acquisition of clinical data. LM: acquisition of clinical data. JS: results interpretation, and manuscript drafting and revision. GT: study concept and design, results interpretation, and manuscript revision.

## Conflict of Interest Statement

Cefaly Technology provided the devices. None of the investigators has any financial interest in Cefaly Technology, but JS is a consultant for this company. The other authors declare no conflict of interest. The study was investigator-initiated and not industry-sponsored.

## References

[1] Global Burden of Disease Study 2013 Collaborators. Global, regional, and national incidence, prevalence, and years lived with disability for 301 acute and chronic diseases and injuries in 188 countries, 1990-2013: a systematic analysis for the Global Burden of Disease Study 2013. Lancet (2015) 386(9995):743–800. 10.1016/S0140-6736(15)60692-4 26063472 PMC4561509

[2] Coppola G Di Lorenzo C Serrao M Parisi V Schoenen J Pierelli F Pathophysiological targets for non-pharmacological treatment of migraine. Cephalalgia (2015). 10.1177/0333102415620908 26637237

[3] Ambrosini A D’Alessio C Magis D Schoenen J Targeting pericranial nerve branches to treat migraine: current approaches and perspectives. Cephalalgia (2015) 35(14):1308–22. 10.1177/0333102415573511 25736180

[4] Schoenen J Vandersmissen B Jeangette S Herroelen L Vandenheede M Gérard P Migraine prevention with a supraorbital transcutaneous stimulator: a randomized controlled trial. Neurology (2013) 80(8):697–704. 10.1212/WNL.0b013e3182825055 23390177

[5] Blumenfeld AM Bloudek LM Becker WJ Buse DC Varon SF Maglinte GA Patterns of use and reasons for discontinuation of prophylactic medications for episodic migraine and chronic migraine: results from the second international burden of migraine study (IBMS-II). Headache (2013) 53(4):644–55. 10.1111/head.12055 23458496

[6] Magis D Sava S d’Elia TS Baschi R Schoenen J. Safety and patients’ satisfaction of transcutaneous supraorbital neurostimulation (tSNS) with the Cefaly^®^ device in headache treatment: a survey of 2,313 headache sufferers in the general population. J Headache Pain (2013) 14:95. 10.1186/1129-2377-14-95 24289825 PMC4177534

[7] Russo A Tessitore A Conte F Marcuccio L Giordano A Tedeschi G Transcutaneous supraorbital neurostimulation in “de novo” patients with migraine without aura: the first Italian experience. J Headache Pain (2015) 16:69 10.1186/1129-2377-16-S1-A136 26197977 PMC4510103

[8] Magis D D’Ostilio K Thibaut A De Pasqua V Gerard P Hustinx R Cerebral metabolism before and after external trigeminal nerve stimulation in episodic migraine. Cephalalgia (2016). 10.1177/0333102416656118 PMC5560481 27342225

[9] Headache Classification Committee of the International Headache Society (IHS). The International Classification of Headache Disorders, 3rd edition (beta version). Cephalalgia (2013) 33(9):629–808. 10.1177/0333102413485658 23771276

[10] Bayliss MS Dewey JE Dunlap I Batenhorst AS Cady R Diamond ML A study of the feasibility of Internet administration of a computerized health survey: the headache impact test (HIT). Qual Life Res (2003) 12(8):953–61. 10.1023/A:1026167214355 14651414

[11] Russo A Tessitore A Esposito F Marcuccio L Giordano A Conforti R Pain processing in patients with migraine: an event-related fMRI study during trigeminal nociceptive stimulation. J Neurol (2012) 259(9):1903–12. 10.1007/s00415-012-6438-1 22349864

[12] Russo A Esposito F Conte F Fratello M Caiazzo G Marcuccio L Functional interictal changes of pain processing in migraine with ictal cutaneous allodynia. Cephalalgia (2017) 37(4):305–314. 10.1177/0333102416644969 27084886

[13] Friston KJ Holmes AP Poline JB Grasby PJ Williams SC Frackowiak RS Analysis of fMRI time-series revisited. Neuroimage (1995) 2(1):45–53. 10.1006/nimg.1995.1007 9343589

[14] Forman SD Cohen JD Fitzgerald M Eddy WF Mintun MA Noll DC. Improved assessment of significant activation in functional magnetic resonance imaging (fMRI): use of a cluster-size threshold. Magn Reson Med (1995) 33(5):636–47. 10.1002/mrm.1910330508 7596267

[15] Schwedt TJ Chiang CC Chong CD Dodick DW. Functional MRI of migraine. Lancet Neurol (2015) 14(1):81–91. 10.1016/S1474-4422(14)70193-0 25496899 PMC11318354

[16] Kerr FW Central relationships of trigeminal and cervical primary afferents in the spinal cord and medulla. Brain Res (1972) 43(2):561–72. 10.1016/0006-8993(72)90408-8 5053289

[17] Villanueva L Noseda R Trigeminal mechanisms of nociception. In: McMahon SB Koltzenburg M Tracey I Turk DC, editors. Wall and Melzack’s Textbook of Pain. Philadelphia: Elsevier Health Sciences (2013). p. 793–802.

[18] Magis D Schoenen J. Advances and challenges in neurostimulation for headaches. Lancet Neurol (2012) 11(8):708–19. 10.1016/S1474-4422(12)70139-4 22814542

[19] Matharu MS Bartsch T Ward N Frackowiak RS Weiner R Goadsby PJ. Central neuromodulation in chronic migraine patients with suboccipital stimulators: a PET study. Brain (2004) 127(Pt 1):220–30. 10.1093/brain/awh022 14607792

[20] Magis D Bruno MA Fumal A Gérardy PY Hustinx R Laureys S Central modulation in cluster headache patients treated with occipital nerve stimulation: an FDG-PET study. BMC Neurol (2011) 11:25. 10.1186/1471-2377-11-25 21349186 PMC3056751

[21] Baliki MN Chialvo DR Geha PY Levy RM Harden RN Parrish TB Chronic pain and the emotional brain: specific brain activity associated with spontaneous fluctuations of intensity of chronic back pain. J Neurosci (2006) 26(47):12165–73. 10.1523/JNEUROSCI.3576-06.2006 17122041 PMC4177069

[22] Becerra L Veggeberg R Prescot A Jensen JE Renshaw P Scrivani S A ‘complex’ of brain metabolites distinguish altered chemistry in the cingulate cortex of episodic migraine patients. Neuroimage Clin (2016) 11:588–94. 10.1016/j.nicl.2016.03.020 27158591 PMC4846856

[23] Hadjipavlou G Dunckley P Behrens TE Tracey I. Determining anatomical connectivities between cortical and brainstem pain processing regions in humans: a diffusion tensor imaging study in healthy controls. Pain (2006) 123(1–2):169–78. 10.1016/j.pain.2006.02.027 16616418

[24] Valet M Sprenger T Boecker H Willoch F Rummeny E Conrad B Distraction modulates connectivity of the cingulo-frontal cortex and the midbrain during pain-an fMRI analysis. Pain (2004) 109(3):399–408. 10.1016/j.pain.2004.02.033 15157701

[25] Tracey I Mantyh PW. The cerebral signature for pain perception and its modulation. Neuron (2007) 55(3):377–91. 10.1016/j.neuron.2007.07.012 17678852

[26] DeSantana JM Da Silva LF De Resende MA Sluka KA. Transcutaneous electrical nerve stimulation at both high and low frequencies activates ventrolateral periaqueductal grey to decrease mechanical hyperalgesia in arthritic rats. Neuroscience (2009) 163(4):1233–41. 10.1016/j.neuroscience.2009.06.056 19576962 PMC3955259

[27] Tessitore A Russo A Esposito F Giordano A Taglialatela G De Micco R Interictal cortical reorganization in episodic migraine without aura: an event-related fMRI study during parametric trigeminal nociceptive stimulation. Neurol Sci (2011) 32(Suppl 1):S165–7. 10.1007/s10072-011-0537-0 21533737

[28] Wager TD Rilling JK Smith EE Sokolik A Casey KL Davidson RJ Placebo-induced changes in FMRI in the anticipation and experience of pain. Science (2004) 303(5661):1162–7. 10.1126/science.1093065 14976306

